# Solid Indeterminate Pulmonary Nodules of Less Than 300 mm^3^: Application of Different Volume Doubling Time Cut-offs in Clinical Practice

**DOI:** 10.3390/diagnostics9020062

**Published:** 2019-06-20

**Authors:** Andrea Borghesi, Silvia Michelini, Alessandra Scrimieri, Salvatore Golemi, Roberto Maroldi

**Affiliations:** 1 Department of Radiology, University and ASST Spedali Civili of Brescia, Piazzale Spedali Civili 1, 25123 Brescia, Italy; ascrimieri@alice.it (A.S.); golemisalvatore@gmail.com (S.G.); roberto.maroldi@unibs.it (R.M.); 2 Department of Radiology, Fondazione Poliambulanza Istituto Ospedaliero, Via Leonida Bissolati, 57, 25124 Brescia, Italy; silviamichelini2015@gmail.com

**Keywords:** solitary pulmonary nodule, lung neoplasm, multidetector computed tomography, computer-assisted image analysis

## Abstract

In the British Thoracic Society guidelines for incidental pulmonary nodules, volumetric analysis has become the recommended method for growth assessment in solid indeterminate pulmonary nodules (SIPNs) <300 mm^3^. In these guidelines, two different volume doubling time (VDT) cut-offs, 400 and 600 days, were proposed to differentiate benign from malignant nodules. The present study aims to evaluate the performance of these VDT cut-offs in a group of SIPNs <300 mm^3^ which were incidentally detected in a routine clinical setting. During a 7-year period, we retrospectively selected 60 patients with a single SIPN <300 mm^3^. For each SIPN, the volume and VDT were calculated using semiautomatic software throughout the follow-up period, and the performance of the 400- and 600-day VDT cut-offs was compared. In the selected sample, there were 38 benign and 22 malignant nodules. In this group of nodules, the sensitivity, negative predictive value and accuracy of the 600-day VDT cut-off were higher than those of the 400-day VDT cut-off. Therefore, in the management of SIPNs <300 mm^3^ which were incidentally detected in a clinical setting, the 600-day VDT cut-off was better at differentiating benign from malignant nodules than the 400-day VDT cut-off, by reducing the number of false negatives.

## 1. Introduction

Pulmonary nodules are a frequent incidental finding in clinical practice [1,2,3]. In fact, with the introduction and broad availability of multidetector row computed tomography (MDCT) scanners and thin-section CT images, an increasing number of pulmonary nodules are being detected, especially those of small size [4].

Small pulmonary nodule is a term that should be reserved for nodules less than 8 mm in size. The malignancy rate of these pulmonary nodules is very low; however, a confident diagnosis of benignity is possible only for calcified, fat-containing and perifissural nodules [5].

Calcification patterns suggesting the benign nature of nodules are diffuse, central, popcorn-like and laminar [1,5]. Other features that suggest benignity are smooth margins and a spherical shape [1,5]. However, it should be noted that in patients with an oncological history, pulmonary metastases may exhibit smooth margins and a spherical shape on CT images. Therefore, the majority of pulmonary nodules less than 8 mm remain indeterminate even if analyzed with thin-section MDCT [6,7].

For these small nodules, contrast-enhanced CT and fluorine-18 (FDG) positron emission tomography (PET) are inaccurate methods for discriminating between benign and malignant lesions. Therefore, the main indicator of malignancy in pulmonary nodules less than 8 mm is growth rate.

In clinical practice, the determination of the growth rate is an important and cost-effective step in the evaluation of small pulmonary nodules. It is generally estimated by comparing nodule size (specifically the maximum or average diameter) on current and prior axial CT images [8]. However, this two-dimensional sizing method has some limitations. The major limitations are the inability to detect the asymmetrical growth of nodules in the longitudinal plane and the low repeatability and reproducibility [9]. To overcome these limitations, some authors recommend that the growth rate of pulmonary nodules be assessed by calculating volumetric changes and the volume doubling time (VDT) with the aid of dedicated three-dimensional software [10,11,12,13]. Although intra- and inter- observer agreement for software-derived volumetric analysis is relatively low for subsolid nodules less than 8 mm [14,15], the repeatability and reproducibility of volumetric analysis for small solid nodules are more consistent and widely reported in the medical literature [14,16,17].

In the recently published British Thoracic Society (BTS) guidelines for incidental pulmonary nodules, volumetric analysis with the VDT calculation has become the recommended method for growth assessment in solid nodules of less than 8 mm or 300 mm^3^ detected in adults (≥18 years), regardless of risk factors (such as smoking habits and oncologic history) [7,18]. In the BTS guidelines, a volumetric increase of more than 25% is considered clinically significant, and a VDT ≤400 days at any time during a follow-up CT scan is considered suspicious for malignancy; therefore, in these cases, definitive management (biopsy or resection) is advocated [7,18]. Conversely, conservative management is recommended for nodules with a VDT >600 days [7,18].

The main purpose of this study was to retrospectively evaluate the performance of the VDT cut-offs of 400 and 600 days at the first and last follow-up CT scans in a group of solitary solid indeterminate pulmonary nodules (SIPNs) <300 mm^3^ which were incidentally detected on routine MDCT scans.

## 2. Materials and Methods

### 2.1. Patient and Nodule Selection

Through a search on the department radiology information system (RIS)/picture archiving and communication system (PACS) between January 2005 and January 2012, all MDCT reports containing findings indicative of SIPNs ≤8 mm in diameter were retrieved. The search revealed a total of 672 patients with one or more SIPNs ≤8 mm in diameter. The study sample was selected according to the following inclusion criteria: (a) solitary SIPN; (b) nodule diameter ≥3 mm; (c) nodule volume <300 mm^3^; (d) two or more unenhanced MDCT scans performed with the same scanner and the same acquisition/reconstruction protocol; (e) thin-section 1 mm lung window images in DICOM format; and (f) histologic diagnosis after surgical resection or follow-up of more than two years.

Patients younger than 18 years and those who had previously had granulomatous disease or who were undergoing steroid or chemotherapy treatment were excluded.

After applying such criteria, a total of 60 patients with single SIPNs <300 mm^3^ were enrolled. The characteristics of these patients (such as age, sex, smoking habits and oncologic history) are listed in Table 1.

### 2.2. Image Acquisition

All patients/SIPNs were scanned on the same MDCT scanner (Somatom Sensation 16; Siemens, Germany) with the following parameters: collimation, 16 × 0.75 mm; beam pitch, 1.0; rotation time, 0.5 s; tube voltage, 120 kVp; and tube current, 180 mAs. The acquisition of the entire thorax was performed in inspiratory apnea without spirometric control. The acquisition volume was reconstructed as 1-mm slice thickness, applying a sharp reconstruction algorithm and a preset lung window.

### 2.3. Image Analysis

For each SIPN, the axial diameters, the volume at baseline and at the first follow-up CT scan and the VDT at the first follow-up were calculated using three-dimensional semiautomatic software (SAT module, classic version, TeraRecon, Inc., Foster City, CA, USA). Segmentation and volumetric analysis were assessed visually and considered successful when the SIPN was completely outlined. The VDTs were automatically generated by the software at the end of segmentation only in cases where a positive volumetric variation was detected.

In SIPNs with two or more follow-up CT scans, the volumetric variation and the VDT at the last follow-up CT scan were also calculated. As recommended in the BTS guidelines, only a volumetric variation of more than 25% was considered significant [7,10].

Prior to the volumetric analysis, visual assessment of the nodule margins (smooth, lobulated or spiculated), position (intraparenchymal or juxta-vascular/pleural) and lobe location of the SIPNs was performed at the baseline CT scan.

The volumetric analysis and visual assessment of the nodule margins, position and lobe location were performed by a radiologist with 14 years of experience in thoracic imaging and 8 years of experience in using volumetric software.

### 2.4. Final Diagnosis

The final diagnosis of benignity or malignancy was based on the histologic examination of the surgical specimen. SIPNs with a follow-up ≥2 years and with VDTs >600 days were also considered benign.

All procedures performed in this study were in accordance with the ethical standard as laid down in the 1975 Helsinki Declaration and its later amendments. The present study was retrospective, and it did not alter the management of the patients; thus, no specific consent was necessary. However, informed consent for the use of personal data was obtained from all patients.

### 2.5. Statistical Analysis

The data are presented as the number (%) or the mean ± standard deviation for normally distributed data or as the median and interquartile range (IQR) for not normally distributed data.

The performance of the VDT cut-offs of 400 and 600 days at the first and last follow-up CT scans was assessed in terms of sensitivity, specificity, positive predictive value (PPV), negative predictive value (NPV) and accuracy for diagnosis of malignancy.

To identify the relationship between nodule outcome and certain independent variables (such as patient smoking and oncologic history, nodule volume, margins, position and lobe location), a chi-square test was used. Multivariate logistic regression was also used to select independent predictive variables of malignancy.

The statistical analysis was performed using dedicated software (MedCalc Software, version 19), and a *p* value <0.05 was considered statistically significant.

## 3. Results

Segmentation and volumetric analysis were successfully performed in all cases. At the baseline MDCT scan, 31/60 (51.7%) SIPNs had a volume <80 mm^3^ (mean, 51.0 ± 13.1 mm^3^), and 29/60 (48.3%) SIPNs had a volume ranging between 80 and 300 mm^3^ (mean, 149.3 ± 63.5 mm^3^). 

The nodule characteristics (size, margins, lobe location and position) at the baseline MDCT scan are listed in Table 2.

Each SIPN was scanned 2–13 times (median, 4 times; IQR, 3–5 times), and in 47/60 (78.3%) SIPNs, two or more follow-up CT scans were available. The time interval between the baseline scan and the first follow-up CT scan ranged from 63 to 446 days (median, 155 days; IQR, 110–220 days). The time interval between the baseline scan and the last follow-up CT scan in SIPNs with two or more follow-ups ranged from 209 to 2744 days (median, 1089 days; IQR, 763–1340 days).

A significant growth (volumetric increment >25% with a VDT <400 days) between the baseline and the first CT scan was observed in 19/60 (31.7%) SIPNs (9 nodules <80 mm^3^ and 10 between 80–300 mm^3^). In these growing nodules, the volume increased from 36 to 465% (median, 126.0%; IQR, 65.3–276.5%), with the VDT ranging from 63 to 379 days (median, 121 days; IQR, 102–180 days). These growing nodules with a histological diagnosis of malignancy (11 metastases and 8 non-small cell lung cancers (NSCLCs)) were surgically removed (Table 3 and Table 4).

**Figure 1:**
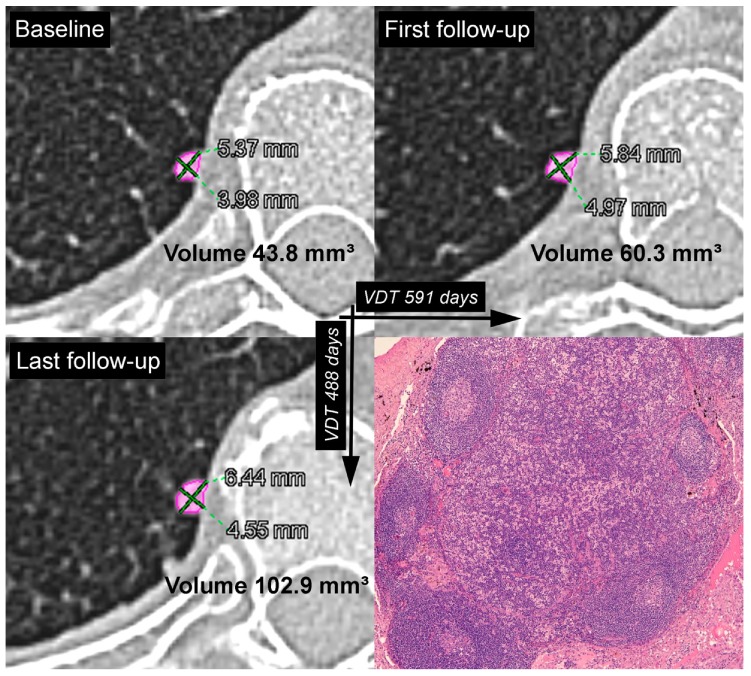
Solid pulmonary nodule with smooth margins located in the right lower lobe of a 57-year-old female patient with oncologic history. Baseline (top left), first follow-up (top right) and last follow-up (bottom left) CT images. The time interval between the baseline and the first follow-up CT scans was 272 days. The time interval between the baseline and the last follow-up CT scan was 601 days. In the first follow-up, the software calculated a relative volume variation of 38% with a VDT of 591 days. The relative volume variation and VDT in the last follow-up were 135% and 488 days, respectively. The nodule was surgically removed, and histologic examination of the specimen revealed an intrapulmonary lymph node (bottom right).

At the first follow-up CT scan, a significant volumetric increment (more than 25%) was observed in 3/60 (5.0%) other SIPNs (1 nodule <80 mm^3^ and 2 nodules between 80–300 mm^3^) (Table 5). In these growing nodules, the VDT ranged from 407 to 591 days (Table 5). Another SIPN with a VDT of 458 days had a volumetric increment of 17%. All these 4 SIPNs were surgically removed (2 NSCLCs, 1 metastasis from colon cancer and 1 benign intrapulmonary lymph node (IPLN)) (Table 4 and Table 5). The metastasis and the IPLN were surgically removed after two consecutive follow-up CT scans. At the first follow-up CT scan (105 days after the baseline scan), the metastasis had a volumetric increment of 17%, with a VDT of 458 days; in the second follow-up (440 days after the baseline scan), the metastasis exhibited a significant volumetric increase (+260% compared to the baseline scan), with a VDT of 238 days. The IPLN had a volumetric increment of 38% with a VDT of 591 days at the first follow-up CT scan (272 days after the baseline scan), and in the second (601 days after the baseline scan), it exhibited a further significant volumetric increase (+135% compared to the baseline scan), with a VDT of 488 days (Figure 1).

None of the remaining 37/60 (61.7%) SIPNs with a follow-up ≥2 years showed significant growth with VDT <600 days at the first and last follow-up CT scans. In these SIPNs, the time interval between the baseline scan and the last follow-up CT scan ranged from 757 to 2744 days (median, 1107 days; IQR, 1058–1446 days), and the VDTs at the last follow-up CT scan were ≥1347 days.

In our sample, assuming a malignant doubling time cut-off of 400 days at the first follow-up CT scan, we obtained a sensitivity, specificity, PPV, NPV and accuracy of 86%, 100%, 100%, 93% and 95%, respectively (Table 6). At the last follow-up CT scan, the VDT cut-off of 400 days had a sensitivity, specificity, PPV, NPV and accuracy of 91%, 100%, 100%, 95% and 97%, respectively (Table 7). In contrast, considering a malignant doubling time cut-off of 600 days, we obtained a sensitivity, specificity, PPV, NPV and accuracy of 100%, 97%, 96%, 100%, and 98%, respectively, at both the first and last follow-up CT scans (Table 6 and Table 7).

The relationships between nodule outcome and independent variables, including patient smoking and oncologic history, nodule volume, margins, position and lobe location, are summarized in Table 8. Oncologic history and nodule margins were significantly associated with the final diagnosis of the pulmonary nodules (*p* ≤ 0.005). Just outside the limits of significance was the relationship between nodule outcome and smoking history (*p* = 0.063).

In the multivariate logistic regression analysis, oncologic history (odds ratio, 5.0; 95% confidence interval, 1.27–19.75; *p* = 0.022) and nodule margins (odds ratio, 6.3; 95% confidence interval, 1.91–20.87; *p* = 0.003) remained significant independent variables for predicting malignancy.

## 4. Discussion

The usefulness of quantitative CT analysis has been widely demonstrated in the literature, and its applications in the thoracic field are constantly expanding [13,19,20,21,22,23]. Among these quantitative CT applications, one of the most well-known and commonly-used in radiology departments is computer-aided nodule volumetry [8,24]. This method provides a more accurate and reproducible measurement of nodules (especially solid nodules) than two-dimensional measurements with electronic calipers, and consequently improves the stratification of nodule malignancy likelihood and the monitoring of indeterminate nodules [8,24,25].

While in many lung cancer screening trials nodule, management and growth determination are based on volume assessment and the VDT calculation [26,27,28,29,30], the differentiation between stable and growing nodules in clinical practice is conventionally performed on two-dimensional images as changes in axial diameters (maximum or average) [6,8].

In contrast, with this standard of care, the BTS guidelines for the management of pulmonary nodules (published in 2015) [7] were the first to recommend nodule volumetry, calculated with three-dimensional software, as the preferred sizing method for risk stratification and growth determination in pulmonary nodules, regardless of their route of detection (lung cancer screening or clinical practice) [7,18].

The new standard of care proposed by the BTS guidelines includes the use of nodule volume and VDT for the management of solid pulmonary nodules, especially for nodules <300 mm^3^ [7,18]. In this statement, the authors define two different VDT cut-offs (400 and 600 days) to improve the assessment of the probability of malignancy and to more appropriately define the follow-up (time intervals and total duration) for these small pulmonary nodules [7,18]. For example, a solid pulmonary nodule <300 mm^3^ with a VDT <400 days (calculated 3 months or 1 year after the baseline CT scan) is considered suspicious for malignancy; therefore, definitive management (such as surgical resection) should be recommended [7,18]. Conversely, a solid pulmonary nodule <300 mm^3^ with a VDT >600 days (calculated 1 year after the baseline scan) is most likely benign, and therefore, the follow-up should be concluded without further investigation [7,18]. For SIPNs with a VDT of 400–600 days, the BTS guidelines suggest continuing yearly surveillance or a biopsy, depending on patient preference [7,18].

To the best of our knowledge, the present study is the first to evaluate the performance of the VDT cut-offs of 400 and 600 days in a group of SIPNs <300 mm^3^ which were incidentally detected in a routine clinical setting. The present study found that in its sample, the diagnostic performance of the VDT cut-off of 600 days in differentiating benign from malignant nodules was better than that of the VDT cut-off of 400 days, at both the first and last follow-up CT scans. With the 600-day VDT cut-off, there would have been 1 false positive and no false negatives (sensitivity and NPV, 100%). In contrast, with the 400-day VDT cut-off, there would have been no false positives but 3 false negatives, reducing the sensitivity and NPV from 100% to 86% and from 100% to 93%, respectively.

The medical literature on SIPNs states that in a lung cancer screening program, a 400-day VDT cut-off is commonly used as the trigger for the referral of patients to a chest physician for further investigation and diagnosis [8,26,28,29,31]. Henschke et al. [32] reported that in their series of 111 lung cancer cases, all 99 malignant solid pulmonary nodules found in CT screening of the International Early Lung Cancer Action Program had VDTs <400 days.

In clinical practice, this cut-off is also accepted as the upper limit of VDT, above which solid nodules could be considered benign [33]. However, Mikita et al. [34] reported that malignant solid nodules sometimes exhibited a slow growth pattern with a VDT >400 days. In line with these data, we found that 3/22 (13.6%) malignant solid nodules <300 mm^3^ exhibited a VDT >400 days (Table 5). These 3 malignant small nodules found in our sample, corresponding to 2 NSCLCs and 1 metastasis, had a software-calculated VDT ranging from 407 to 458 days at the first follow-up CT scan (Table 5). However, at the same follow-up CT scan, the 2 NSCLCs showed significant volume increases of 71 and 75% (Table 5). Moreover, the metastasis had a nonuniform growth rate, and at the last follow-up CT scan, it exhibited a significant increase in volume (+260% compared to the baseline scan), with a VDT of 238 days.

On the other hand, our study found 1 fast-growing benign nodule with a significant increase in volume (+135% compared to the baseline scan), and a software-calculated VDT of 488 days at the last follow-up CT scan (Figure 1). On the basis of this significant growth, this nodule was considered suggestive of a malignant lesion and was surgically removed. Histological examination of the specimen revealed an IPLN (Figure 1).

IPLNs are not uncommon lesions, and are usually detected along the subpleural lymphatic chain below the level of the carina [35]. Moreover, as noted in our case, some authors have reported that IPLNs may increase in size over time, and thus be confused with metastatic or primary lung cancer [35].

The present study also found that in the multivariate analysis, only the oncologic history and nodule margins were significant independent variables for predicting malignancy.

This study has some limitations. First, it was retrospectively performed. Second, a relatively small number of SIPNs were evaluated; however, the inclusion criteria were very strict, and only patients with a single SIPN <300 mm^3^ were selected. Third, the volumetric analysis and visual assessment of the nodule characteristics were performed by one observer; however, his experience in thoracic imaging and nodule volumetry may have improved the accuracy of the analysis.

## 5. Conclusions

This retrospective study noted that in SIPNs <300 mm^3^, the 600-day VDT cut-off was better at differentiating benign from malignant nodules than the 400-day VDT cut-off by reducing the number of false negatives. In addition, we found that oncologic history and nodule margins were also significant parameters for predicting malignancy.

## Figures and Tables

**Table 1:** Characteristics of the study patients (*n* = 60).

Characteristics	
Age (years)	62.9 ± 11.7
Sex	
Male	38 (63.3)
Female	22 (36.7)
Smoking habits	
Current/former	26 (43.3)
Never	34 (56.7)
Oncologic history	32 (53.3)
Colorectal cancer	6 (18.8)
Non-small cell lung cancer	5 (15.6)
Melanoma	5 (15.6)
Other malignancy	16 (50)

Data are presented as numbers (%) or means ± standard deviations.

**Table 2:** Characteristics of the 60 SIPNs <300 mm^3^ on baseline MDCT.

Characteristics	SIPNs
Volume	
<80 mm^3^	31 (51.7)
80–300 mm^3^	29 (48.3)
Margins	
Smooth	43 (71.7)
Lobulated	10 (16.7)
Spiculated	7 (11.7)
Position	
Juxta-vascular/pleural	17 (28.3)
Intra-parenchymal	43 (71.7)
Lobe location	
Middle/Lower	33 (55.0)
Upper	27 (45.0)

Data are presented as numbers (%).

**Table 3:** Patient risk status and histological features of the 19 SIPNs with VDT <400 days.

Histology	Patient Risk Status		
	Oncologic History	Oncologic and Smoking History	Smoking History
Metastasis	9 (81.8)	2 (18.2)	-
NSCLC	-	5 (62.5)	3 (37.5)

Data are presented as numbers (%). NSCLC, non-small cell lung cancer.

**Table 4:** Histological features and surgical treatment of the resected SIPNs (*n* = 23).

Histology	Type of Surgical Procedure				
	Wedge Resection	Segmentectomy	Lobectomy	Lymph Node Sampling	Lymph Node Dissection
Metastasis	12 (100)	-	-	1 (8.3) *	-
NSCLC	4 (40)	5 (50)	1 (10)	6 (60) *	1 (10) *
IPLN	1 (100)	-	-	-	-

Data are presented as numbers (%). NSCLC, non-small cell lung cancer. IPLN, intrapulmonary lymph node. * No lymph node metastases.

**Table 5:** Characteristics and histological features of the 4 SIPNs with VDT between 400–600 days.

Nodule	Patient Risk Status	Volume * (mm^3^)	Margins *	Lobe Location	Time Interval 1^st^–2^nd^ CT (days)	Volume Increment ^§^ (%)	VDT ^§^ (days)	Histology
1	Smoking history	135	Spiculated	Upper	316	71	407	NSCLC
2	Smoking history	107	Lobulated	Lower	360	75	447	NSCLC
3	Oncologic history	69	Spiculated	Upper	105	17	458	Metastasis
4	Oncologic history	44	Smooth	Lower	272	38	591	IPLN

* At the baseline CT; ^§^ At the first CT follow-up; VDT, volume doubling time; NSCLC, non-small cell lung cancer; IPLN, intrapulmonary lymph node.

**Table 6:** Performance of the volume doubling time cut-offs of 400 and 600 days at the first follow-up.

First Follow-up CT Scan	Nodule Outcome		SE (%)	SP (%)	PPV (%)	NPV (%)	ACC (%)
	Benign (*n* = 38)	Malignant (*n* = 22)					
Volume doubling time			**86**	**100**	100	93	95
≤400 days	-	19					
>400 days	38	3					
Volume doubling time			100	97	96	100	98
≤600 days	1	22					
>600 days	37	-					

Data are presented as numbers. SE, sensitivity; SP, specificity; PPV, positive predictive value; NPV, negative predictive value; ACC, accuracy.

**Table 7:** Performance of the volume doubling time cut-offs of 400 and 600 days at the last follow-up.

Last Follow-up CT Scan	Nodule Outcome		SE (%)	SP (%)	PPV (%)	NPV (%)	ACC (%)
	Benign (*n* = 38)	Malignant (*n* = 22)					
Volume doubling time			91	100	100	95	97
≤400 days	-	20					
>400 days	38	2					
Volume doubling time			100	97	96	100	98
≤600 days	1	22					
>600 days	37	-					

Data are presented as numbers. SE, sensitivity; SP, specificity; PPV, positive predictive value; NPV, negative predictive value; ACC, accuracy.

**Table 8:** Association between nodule outcome and independent variables (smoking and oncologic history, nodule volume, margins, position and lobe location).

Independent Variables	Nodule Outcome		Chi-square Test
	Benign (*n* = 38)	Malignant (*n* = 22)	*p* Value
Smoking history			0.063
No	25 (41.7)	9 (15.0)	
Yes	13 (21.7)	13 (21.7)	
Oncologic history			0.005
No	23 (38.3)	5 (8.3)	
Yes	15 (25.0)	17 (28.3)	
Volume *			0.467
<80 mm^3^	21 (35.0)	10 (16.7)	
80–300 mm^3^	17 (28.3)	12 (20.0)	
Margins *			<0.001 ^†^
Smooth	33 (55.0)	10 (16.7)	
Lobulated	5 (8.3)	5 (8.3)	
Spiculated	-	7 (11.7)	
Position *			0.188
Juxta-vascular/pleural	13 (21.7)	4 (6.7)	
Intra-parenchymal	25 (41.7)	18 (30.0)	
Lobe location *			0.557
Middle/Lower	22 (36.7)	11 (18.3)	
Upper	16 (26.7)	11 (18.3)	

Data are presented as numbers (%). * At the baseline CT. **^†^** Chi-squared test for trend.
